# Supplementary material for: Hormone Replacement Therapy and Risks of Various Cancers in Postmenopausal Women with De Novo or a History of Endometriosis
Source: Cancers (Basel). 2024 Feb 16;16(4):809. doi: 10.3390/cancers16040809 (PMC10886569; doi:10.3390/cancers16040809)
Supplement: Supplementary file 1 [file cancers-16-00809-s001.zip › Table S2.pdf]

Table S2. Characteristics of the dataset for each cancer according to HRT in women with postmenopausal endometriosis (HIRA claims data 2008–2022).

A. Dataset for cervical cancer.

	Total n = 29,412 (100.0%)	HRT (-) n = 14,706 (50.0%)	HRT (+) n = 14,706 (50.0%)	OR (95% CI)	*P* value [a]
Age at last clinic visit (years)	55.5 ± 4.9	55.5 ± 4.9	55.5 ± 4.9	1.000 (0.995, 1.005)	1.000
SES at last clinic visit					
Mid- or high-SES	28,654 (97.4)	14,353 (97.6)	14,301 (97.3)	ref	
Low SES	758 (2.6)	353 (2.4)	405 (2.8)	1.152 (0.997, 1.331)	0.056
CCI at last clinic visit					
0	15,680 (53.3)	8,081 (55.0)	7,599 (51.7)	ref	
1	7,068 (24.0)	3,348 (22.8)	3,720 (25.3)	1.182 (1.117, 1.25)	<0.001
2	3,709 (12.6)	1,815 (12.3)	1,894 (12.9)	1.11 (1.033, 1.192)	0.004
3	1,582 (5.4)	768 (5.2)	814 (5.5)	1.127 (1.016, 1.25)	0.023
Over 4	1,373 (4.7)	694 (4.7)	679 (4.6)	1.040 (0.932, 1.162)	0.481
Age at endometriosis diagnosis (years)	48.4 ± 6.2	48.8 ± 6.1	48.0 ± 6.2	0.979 (0.975, 0.982)	<0.001
Year of endometriosis diagnosis					
2008	1,980 (6.7)	826 (5.6)	1,154 (7.9)	ref	
2009	1,866 (6.3)	855 (5.1)	1,011 (6.9)	0.846 (0.745, 0.962)	0.010
2010	1,923 (6.5)	831 (5.7)	1,092 (7.4)	0.941 (0.828, 1.068)	0.344
2011	1,663 (5.7)	759 (5.2)	904 (6.2)	0.853 (0.748, 0.972)	0.017
2012	1,538 (5.2)	725 (4.9)	813 (5.5)	0.803 (0.702, 0.918)	0.001
2013	1,827 (6.2)	877 (6.0)	950 (6.5)	0.775 (0.682, 0.881)	<0.001
2014	2,162 (7.4)	1031 (7.0)	1,131 (7.7)	0.782 (0.694, 0.888)	<0.001
2015	1,801 (6.1)	907 (6.2)	894 (6.1)	0.706 (0.620, 0.802)	<0.001
2016	1,703 (5.8)	881 (6.0)	822 (5.6)	0.668 (0.586, 0.761)	<0.001
2017	2,510 (8.5)	1,294 (8.8)	1,216 (8.3)	0.673 (0.597, 0.758)	<0.001
2018	2,975 (10.1)	1,568 (10.7)	1,407 (9.6)	0.642 (0.573, 0.720)	<0.001
2019	2,431 (8.3)	1,276 (8.7)	1,155 (7.9)	0.648 (0.575, 0.730)	<0.001
2020	2,088 (7.1)	1,131 (7.7)	957 (6.5)	0.606 (0.535, 0.686)	<0.001
2021	1,748 (5.9)	1,011 (6.9)	737 (5.0)	0.522 (0.458, 0.594)	<0.001
2022	1,197 (4.1)	734 (5.0)	463 (3.2)	0.452 (0.39, 0.523)	<0.001
Hysterectomy for benign disease	9,239 (31.4)	4,918 (33.4)	4,321 (29.4)	0.828 (0.788, 0.870)	<0.001
Methods of surgery for endometriosis					
Ovarian cystectomy	18,366 (62.4)	9,014 (61.3)	9,352 (63.6)	1.103 (1.052, 1.156)	<0.001
BSO or USO	3,630 (12.3)	1,758 (12.0)	1,872 (12.7)	1.074 (1.002, 1.152)	0.043
Hysterectomy	9,266 (31.5)	4,938 (33.6)	4,328 (29.4)	0.825 (0.785, 0.867)	0.000
Fulguration	966 (3.3)	451 (3.1)	515 (3.5)	1.147 (1.009, 1.304)	0.036
Number of surgery for endometriosis					
1	28,382 (96.5)	14,303 (97.3)	14,079 (95.7)	ref	
2	986 (3.4)	389 (2.6)	597 (4.1)	1.559 (1.369, 1.775)	<0.001
Over 3	44 (0.2)	14 (0.1)	30 (0.2)	2.177 (1.154, 4.106)	0.016
Time between endometriosis diagnosis and beginning of HRT					
Rate of HRT use before endometriosis diagnosis			3,835 (26.1)		
Time of HRT use before endometriosis diagnosis (years)			0.7 ± 1.7		
Time of HRT use after endometriosis diagnosis (years)			1.7 ± 2.1		
Rate of HRT use after endometriosis diagnosis			10,871 (73.9)		
Time of HRT use after endometriosis diagnosis (years)			1.2 ± 1.9		

CI, confidence interval; HIRA, Health Insurance Review & Assessment Service; HRT, hormone replacement therapy; OR, odds ratio.
All values are expressed as mean ± standard deviation or number (%).
[a] Univariate logistic regression

B. Dataset for uterine cancer.

	Total n = 29,294 (100.0%)	HRT (-) n = 14,647 (50.0%)	HRT (+) n = 14,647 (50.0%)	OR (95% CI)	*P* value [a]
Age at last clinic visit (years)	55.5 ± 4.9	55.5 ± 4.9	55.5 ± 4.9	1.000 (0.995, 1.005)	1.000
SES at last clinic visit					
Mid- or high-SES	28,523 (97.4)	14,284 (97.5)	14,239 (97.2)	ref	
Low SES	771 (2.6)	363 (2.5)	408 (2.8)	1.128 (0.977, 1.301)	0.101
CCI at last clinic visit					
0	15,693 (53.6)	8,113 (55.4)	7,580 (51.8)	ref	
1	6,994 (23.9)	3,288 (22.5)	3,706 (25.3)	1.206 (1.140, 1.276)	<0.001
2	3,721 (12.7)	1,837 (12.5)	1,884 (12.9)	1.098 (1.022, 1.179)	0.011
3	1,549 (5.3)	751 (5.1)	798 (5.5)	1.137 (1.025, 1.263)	0.016
Over 4	1,337 (4.6)	658 (4.5)	679 (4.6)	1.105 (0.988, 1.235)	0.081
Age at endometriosis diagnosis (years)	48.4 ± 6.1	48.8 ± 6.1	48.0 ± 6.2	0.978 (0.974, 0.982)	<0.001
Year of endometriosis diagnosis					
2008	1,994 (6.8)	841 (5.7)	1,153 (7.9)	ref	
2009	1,861 (6.4)	849 (5.8)	1,012 (6.9)	0.869 (0.766, 0.988)	0.031
2010	1,909 (6.5)	814 (5.6)	1,095 (7.5)	0.981 (0.864, 1.114)	0.77
2011	1,682 (5.7)	775 (5.3)	907 (6.2)	0.854 (0.749, 0.973)	0.018
2012	1,491 (5.1)	680 (4.6)	811 (5.5)	0.87 (0.76, 0.996)	0.043
2013	1,832 (6.3)	879 (6)	953 (6.5)	0.791 (0.7, 0.899)	<0.001
2014	2,133 (7.3)	1,004 (6.9)	1,129 (7.7)	0.820 (0.725, 0.928)	0.002
2015	1,830 (6.3)	936 (6.4)	894 (6.1)	0.697 (0.613, 0.792)	<0.001
2016	1,716 (5.9)	895 (6.1)	821 (5.6)	0.669 (0.588, 0.762)	<0.001
2017	2,509 (8.6)	1,294 (8.8)	1,215 (8.)	0.685 (0.608, 0.771)	<0.001
2018	2,971 (10.1)	1,568 (10.7)	1,403 (9.6)	0.653 (0.582, 0.732)	<0.001
2019	2,448 (8.4)	1,302 (8.9)	1,146 (7.8)	0.642 (0.57, 0.723)	<0.001
2020	2,035 (7.0)	1,098 (7.5)	937 (6.4)	0.622 (0.55, 0.705)	<0.001
2021	1,743 (6.0)	1,026 (7)	717 (4.9)	0.51 (0.447, 0.581)	<0.001
2022	1,140 (3.9)	686 (4.7)	454 (3.1)	0.483 (0.416, 0.56)	<0.001
Hysterectomy for benign disease	9,115 (31.1)	4,860 (33.2)	4,255 (29.1)	0.825 (0.785, 0.866)	<0.001
Methods of surgery for endometriosis					
Ovarian cystectomy	18,264 (62.4)	8,948 (61.1)	9,316 (63.6)	1.113 (1.062,1.167)	<0.001
BSO or USO	3,650 (12.5)	1,777 (12.1)	1,873 (12.8)	1.062 (0.991,1.138)	0.09
Hysterectomy	9,187 (31.4)	4,904 (33.5)	4,283 (29.2)	0.821 (0.781,0.863)	<0.001
Fulguration	971 (3.3)	456 (3.1)	515 (3.5)	1.134 (0.998, 1.289)	0.054
Number of surgery for endometriosis					
1	28,277 (96.5)	14,261 (97.4)	14,016 (95.7)	ref	
2	974 (3.3)	376 (2.6)	598 (4.1)	1.618 (1.419, 1.845)	<0.001
Over 3	43 (0.2)	10 (0.1)	33 (0.2)	3.358 (1.655, 6.811)	0.001
Time between endometriosis diagnosis and beginning of HRT					
Rate of HRT use before endometriosis diagnosis			3,794 (25.9)		
Time of HRT use before endometriosis diagnosis (years)			0.7 ± 1.6		
Time of HRT use after endometriosis diagnosis (years)			1.7 ± 2.1		
Rate of HRT use after endometriosis diagnosis			10,853 (74.1)		
Time of HRT use after endometriosis diagnosis (years)			1.3 ± 1.9		

CI, confidence interval; HIRA, Health Insurance Review & Assessment Service; HRT, hormone replacement therapy; OR, odds ratio.
All values are expressed as mean ± standard deviation or number (%).
[a] Univariate logistic regression

C. Dataset for ovarian cancer.

	Total n = 29,448 (100.0%)	HRT (-) n = 14,724 (50.0%)	HRT (+) n = 14,724 (50.0%)	OR (95% CI)	*P* value [a]
Age at last clinic visit (years)	55.5 ± 4.9	55.5 ± 4.9	55.5 ± 4.9	1.000 (0.995, 1.005)	1.000
SES at last clinic visit					
Mid- or high-SES	28,701 (97.5)	14,386 (97.7)	14,315 (97.2)	ref	
Low SES	747 (2.5)	338 (2.3)	409 (2.8)	1.216 (1.051, 1.407)	0.009
CCI at last clinic visit					
0	15,644 (53.1)	8,055 (54.7)	7,589 (51.5)	ref	
1	7,109 (24.1)	3,386 (23.0)	3,723 (25.3)	1.167 (1.103, 1.234)	<0.001
2	3,737 (12.7)	1,841 (12.5)	1,896 (12.9)	1.093 (1.018, 1.174)	0.015
3	1,573 (5.3)	750 ((5.2)	823 (5.6)	1.165 (1.05, 1.292)	0.004
Over 4	1,385 (4.7)	692 (4.7)	693 (4.7)	1.063 (0.952, 1.186)	0.276
Age at endometriosis diagnosis (years)	48.5 ± 6.2	48.9 ± 6.1	48.0 ± 6.2	0.979 (0.975, 0.982)	<0.001
Year of endometriosis diagnosis					
2008	1,970 (6.7)	818 (5.6)	1,152 (7.8)	ref	
2009	1,886 (6.4)	879 (6.0)	1,007 (6.8)	0.814 (0.716, 0.924)	0.002
2010	1,888 (6.4)	793 (5.4)	1,095 (7.4)	0.981 (0.863, 1.114)	0.763
2011	1,697 (5.8)	795 (5.4)	902 (6.1)	0.806 (0.707, 0.918)	0.001
2012	1,513 (5.1)	700 (4.8)	813 (5.5)	0.825 (0.721, 0.94)	0.005
2013	1,841 (6.3)	895 (6.1)	946 (6.4)	0.751 (0.660, 0.853)	<0.001
2014	2,137 (7.3)	1,009 (6.9)	1,128 (7.7)	0.794 (0.702, 0.898)	<0.001
2015	1,796 (6.1)	905 (6.2)	891 (6.1)	0.699 (0.615, 0.795)	<0.001
2016	1,662 (5.6)	843 (5.7)	819 (5.6)	0.69 (0.605, 0.787)	<0.001
2017	2,527 (8.6)	1,311 (8.9)	1,216 (8.3)	0.659 (0.585, 0.742)	<0.001
2018	3,047 (10.4)	1,619 (11)	1,428 (9.7)	0.626 (0.559, 0.702)	<0.001
2019	2,469 (8.4)	1,307 (8.9)	1,162 (7.9)	0.631 (0.560, 0.711)	<0.001
2020	2,070 (7.0)	1,105 (7.5)	965 (6.6)	0.620 (0.548, 0.702)	<0.001
2021	1,763 (6.0)	1,032 (7.0)	731 (5.0)	0.503 (0.442, 0.573)	<0.001
2022	1,182 (4.0)	713 (4.8)	469 (3.2)	0.467 (0.403, 0.541)	<0.001
Hysterectomy for benign disease	9,307 (31.5)	4,931 (33.4)	4,376 (29.7)	0.84 (0.8, 0.882)	<0.001
Methods of surgery for endometriosis					
Ovarian cystectomy	18,344 (62.3)	9,017 (61.2)	9,327 (63.4)	1.094 (1.043, 1.147)	<0.001
BSO or USO	3,637 (12.4)	1,765 (12.0)	1,872 (12.7)	1.07 (0.998, 1.146)	0.058
Hysterectomy	9,388 (31.9)	5,016 (34.1)	4,372 (29.7)	0.817 (0.778, 0.859)	<0.001
Fulguration	978 (3.3)	461 (3.1)	517 (3.5)	1.126 (0.991, 1.279)	0.069
Number of surgery for endometriosis					
1	28,434 (96.6)	14,332 (97.3)	14,102 (95.8)	ref	
2	975 (3.3)	382 (2.6)	593 (4.0)	1.578 (1.384, 1.798)	<0.001
Over 3	39 (0.1)	10 (0.1)	29 (0.2)	2.947 (1.437, 6.046)	0.003
Time between endometriosis diagnosis and beginning of HRT					
Rate of HRT use before endometriosis diagnosis			3,858 (26.1)		
Time of HRT use before endometriosis diagnosis (years)			0.7 ± 1.7		
Time of HRT use after endometriosis diagnosis (years)			1.7 ± 2.1		
Rate of HRT use after endometriosis diagnosis			10,901 (73.9)		
Time of HRT use after endometriosis diagnosis (years)			1.3 ± 1.9		

CI, confidence interval; HIRA, Health Insurance Review & Assessment Service; HRT, hormone replacement therapy; OR, odds ratio.
All values are expressed as mean ± standard deviation or number (%).
[a] Univariate logistic regression

D. Dataset for breast cancer.

	Total n = 29,150 (100.0%)	HRT (-) n = 14,575 (50.0%)	HRT (+) n = 14,575 (50.0%)	OR (95% CI)	*P* value [a]
Age at last clinic visit (years)	55.5 ± 4.9	55.5 ± 4.9	55.5 ± 4.9	1.000 (0.995, 1.005)	1.000
SES at last clinic visit					
Mid- or high-SES	28,397 (97.4)	14,224 (97.6)	14,173 (97.2)	ref	
Low SES	753 (2.6)	351 (2.4)	402 (2.8)	1.149 (0.994, 1.329)	0.06
CCI at last clinic visit					
0	15,670 (53.8)	8,141 (55.9)	7,529 (51.7)	ref	
1	7,081 (24.3)	3,372 (23.1)	3,709 (25.5)	1.189 (1.124, 1.258)	<0.001
2	3,628 (12.5)	1,765 (12.1)	1,863 (12.8)	1.141 (1.062, 1.227)	<0.001
3	1,472 (5.1)	676 (4.6)	796 (5.5)	1.273 (1.144, 1.417)	<0.001
Over 4	1,299 (4.5)	621 (4.3)	678 (4.7)	1.181 (1.054, 1.322)	0.004
Age at endometriosis diagnosis (years)	48.4 ± 6.2	48.8 ± 6.1	48.0 ± 6.2	0.979 (0.975, 0.982)	<0.001
Year of endometriosis diagnosis					
2008	1,971 (6.8)	818 (5.6)	1,153 (7.9)	ref	
2009	1,858 (6.4)	852 (5.9)	1,006 (6.9)	0.838 (0.737, 0.952)	0.007
2010	1,901 (6.5)	812 (5.6)	1,089 (7.5)	0.952 (0.838, 1.081)	0.445
2011	1,672 (5.7)	768 (5.3)	904 (6.2)	0.835 (0.732, 0.952)	0.007
2012	1,527 (5.2)	720 (4.9)	807 (5.5)	0.795 (0.695, 0.91)	0.001
2013	1,808 (6.2)	865 (5.9)	943 (6.5)	0.773 (0.680, 0.88)	<0.001
2014	2,147 (7.4)	1,020 (7)	1,127 (7.7)	0.784 (0.693, 0.887)	<0.001
2015	1,791 (6.1)	904 (6.2)	887 (6.1)	0.696 (0.612, 0.792)	<0.001
2016	1,693 (5.8)	887 (6.1)	806 (5.5)	0.645 (0.566, 0.735)	<0.001
2017	2,530 (8.7)	1,322 (9.1)	1,208 (8.3)	0.648 (0.576, 0.730)	<0.001
2018	2,940 (10.1)	1,540 (10.6)	1,400 (9.6)	0.645 (0.575, 0.724)	<0.001
2019	2,399 (8.2)	1,262 (8.7)	1,137 (7.8)	0.639 (0.567, 0.721)	<0.001
2020	2,063 (7.1)	1,120 (7.7)	943 (6.5)	0.597 (0.527, 0.677)	<0.001
2021	1,706 (5.9)	990 (6.8)	716 (4.9)	0.513 (0.45, 0.585)	<0.001
2022	1,144 (3.9)	695 (4.8)	449 (3.1)	0.458 (0.395, 0.532)	<0.001
Hysterectomy for benign disease	9,132 (31.3)	4,857 (33.3)	4,275 (29.3)	0.830 (0.790, 0.873)	<0.001
Methods of surgery for endometriosis					
Ovarian cystectomy	18,156 (62.3)	8,901 (61.1)	9,255 (63.5)	1.109 (1.058, 1.163)	<0.001
BSO or USO	3,646 (12.5)	1,788 (12.3)	1,858 (12.8)	1.045 (0.975, 1.120)	0.215
Hysterectomy	9,139 (31.4)	4,862 (33.4)	4,277 (29.3)	0.83 (0.79, 0.872)	<0.001
Fulguration	967 (3.3)	459 (3.2)	508 (3.5)	1.111 (0.977, 1.263)	0.109
Number of surgery for endometriosis					
1	28,121 (96.5)	14,169 (97.2)	13,952 (95.7)	ref	
2	980 (3.4)	390 (2.7)	590 (4)	1.536 (1.349, 1.75)	<0.001
Over 3	49 (0.2)	16 (0.1)	33 (0.2)	2.095 (1.153, 3.806)	0.015
Time between endometriosis diagnosis and beginning of HRT					
Rate of HRT use before endometriosis diagnosis			3,794 (26.0)		
Time of HRT use before endometriosis diagnosis (years)			0.7 ± 1.7		
Time of HRT use after endometriosis diagnosis (years)			1.7 ± 2.1		
Rate of HRT use after endometriosis diagnosis			1,0781 (74.0)		
Time of HRT use after endometriosis diagnosis (years)			1.3 ±1.9		

CI, confidence interval; HIRA, Health Insurance Review & Assessment Service; HRT, hormone replacement therapy; OR, odds ratio.
All values are expressed as mean ± standard deviation or number (%).
[a] Univariate logistic regression

E. Dataset for colon cancer.

	Total n = 29,470 (100.0%)	HRT (-) n = 14,735 (50.0%)	HRT (+) n = 14,735 (50.0%)	OR (95% CI)	*P* value [a]
Age at last clinic visit (years)	55.5 ± 4.9	55.5 ± 4.9	55.5 ± 4.9	1.000 (0.995, 1.005)	1.000
SES at last clinic visit					
Mid- or high-SES	28,718 (97.5)	14,392 (97.7)	14,326 (97.2)	ref	
Low SES	752 (2.6)	343 (2.3)	409 (2.8)	1.198 (1.036, 1.385)	0.015
CCI at last clinic visit					
0	15,729 (53.4)	8,119 (55.1)	7,610 (51.7)	ref	
1	7,034 (23.9)	3,309 (22.5)	3,725 (25.3)	1.201 (1.135, 1.271)	<0.001
2	3,759 (12.8)	1,861 (12.6)	1,898 (12.9)	1.088 (1.013, 1.168)	0.020
3	1,571 (5.3)	750 (5.1)	821 (5.6)	1.168 (1.053, 1.296)	0.003
Over 4	1,377 (4.7)	696 (4.7)	681 (4.6)	1.044 (0.935, 1.166)	0.445
Age at endometriosis diagnosis (years)	48.4 ± 6.2	48.8 ± 6.1	48.1 ± 6.2	0.979 (0.975, 0.983)	<0.001
Year of endometriosis diagnosis					
2008	2,012 (6.8)	857 (5.8)	1,155 (7.8)	ref	
2009	1,876 (6.4)	862 (5.9)	1,014 (6.9)	0.873 (0.769, 0.991)	0.035
2010	1,940 (6.6)	842 (5.7)	1,098 (7.5)	0.968 (0.853, 1.098)	0.608
2011	1,666 (5.7)	762 (5.2)	904 (6.1)	0.880 (0.772, 1.003)	0.056
2012	1,507 (5.1)	695 (4.7)	812 (5.5)	0.867 (0.758, 0.992)	0.037
2013	1,822 (6.2)	872 (5.9)	950 (6.5)	0.808 (0.712, 0.918)	0.001
2014	2,160 (7.3)	1,029 (7.0)	1,131 (7.7)	0.816 (0.722, 0.922)	0.001
2015	1,814 (6.2)	924 (6.3)	890 (6.0)	0.715 (0.629, 0.812)	<0.001
2016	1,682 (5.7)	869 (5.9)	813 (5.5)	0.694 (0.609, 0.791)	<0.001
2017	2,530 (8.6)	1,307 (8.9)	1,223 (8.3)	0.694 (0.617, 0.781)	<0.001
2018	2,976 (10.1)	1,553 (10.5)	1,423 (9.7)	0.68 (0.607, 0.762)	<0.001
2019	2,472 (8.4)	1,313 (8.9)	1,159 (7.9)	0.655 (0.582, 0.737)	<0.001
2020	2,107 (7.2)	1,143 (7.8)	964 (6.5)	0.626 (0.553, 0.708)	<0.001
2021	1,751 (5.9)	1,015 (6.9)	736 (5.0)	0.538 (0.473, 0.613)	<0.001
2022	1,155 (3.9)	692 (4.7)	463 (3.1)	0.496 (0.429, 0.575)	<0.001
Hysterectomy for benign disease	9,315 (31.6)	4,952 (33.6)	4,363 (29.6)	0.831 (0.791, 0.873)	<0.001
Methods of surgery for endometriosis					
Ovarian cystectomy	18,356 (62.3)	9,023 (61.2)	9,333 (63.3)	1.094 (1.043,1.147)	<0.001
BSO or USO	3,635 (12.3)	1,761 (12.0)	1,874 (12.7)	1.074 (1.002,1.151)	0.045
Hysterectomy	9,317 (31.6)	4,952 (33.6)	4,365 (29.6)	0.832 (0.792,0.874)	<0.001
Fulguration	970 (3.3)	453 (3.1)	517 (3.5)	1.146 (1.008,1.303)	0.037
Number of surgery for endometriosis					
1	28,446 (96.5)	14,343 (97.3)	14,103 (95.7)	ref	
2	979 (3.3)	379 (2.6)	600 (4.1)	1.610 (1.413, 1.835)	<0.001
Over 3	45 (0.2)	13 (0.1)	32 (0.2)	2.503 (1.314, 4.769)	0.005
Time between endometriosis diagnosis and beginning of HRT					
Rate of HRT use before endometriosis diagnosis			3,851 (26.1)		
Time of HRT use before endometriosis diagnosis (years)			0.7 ± 1.7		
Time of HRT use after endometriosis diagnosis (years)			1.7 ± 2.1		
Rate of HRT use after endometriosis diagnosis			10,862 (73.8)		
Time of HRT use after endometriosis diagnosis (years)			1.3 ± 1.9		

CI, confidence interval; HIRA, Health Insurance Review & Assessment Service; HRT, hormone replacement therapy; OR, odds ratio.
All values are expressed as mean ± standard deviation or number (%).
[a] Univariate logistic regression

F. Dataset for gastric cancer.

	Total n = 29,518 (100.0%)	HRT (-) n = 14,759 (50.0%)	HRT (+) n = 14,759 (50.0%)	OR (95% CI)	*P* value [a]
Age at last clinic visit (years)	55.5 ± 4.9	55.5 ± 4.9	55.5 ± 4.9	1.000 (0.995, 1.005)	1.000
SES at last clinic visit					
Mid- or high-SES	28,735 (97.4)	14,385 (97.5)	14,350 (97.2)	ref	
Low SES	783 (2.7)	374 (2.5)	409 (2.8)	0.978 (0.975, 0.982)	<0.001
CCI at last clinic visit					
0	15,656 (53.0)	8,045 (54.5)	7,611 (51.6)	ref	
1	7,107 (24.1)	3,376 (22.9)	3,731 (25.3)	1.168 (1.104, 1.236)	<0.001
2	3,763 (12.8)	1,855 (12.6)	1,908 (12.9)	1.087 (1.013, 1.167)	0.021
3	1,597 (5.4)	778 (5.3)	819 (5.6)	1.113 (1.004, 1.234)	0.042
Over 4	1,395 (4.7)	705 (4.8)	690 (4.7)	1.035 (0.927, 1.154)	0.544
Age at endometriosis diagnosis (years)	48.5 ± 6.2	48.9 ± 6.1	48.0 ± 6.2	0.978 (0.975, 0.982)	<0.001
Year of endometriosis diagnosis					
2008	1,975 (6.7)	817 (5.5)	1,158 (7.9)	ref	
2009	1,887 (6.4)	873 (5.9)	1,014 (6.9)	0.82 (0.722, 0.931)	0.002
2010	1,922 (6.5)	826 (5.6)	1,096 (7.4)	0.936 (0.824, 1.063)	0.309
2011	1,670 (5.7)	766 (5.2)	904 (6.1)	0.833 (0.730, 0.95)	0.006
2012	1,506 (5.1)	696 (4.7)	810 (5.5)	0.821 (0.717, 0.94)	0.004
2013	1,824 (6.2)	875 (5.9)	949 (6.4)	0.765 (0.673, 0.87)	<0.001
2014	2,168 (7.3)	1,035 (7.0)	1,133 (7.7)	0.772 (0.68, 0.873)	<0.001
2015	1,826 (6.2)	933 (6.3)	893 (6.1)	0.675 (0.594, 0.768)	<0.001
2016	1,700 (5.8)	877 (5.9)	823 (5.6)	0.662 (0.581, 0.755)	<0.001
2017	2,488 (8.4)	1,265 (8.6)	1,223 (8.3)	0.682 (0.606, 0.768)	<0.001
2018	3,047 (10.3)	1,624 (11)	1,423 (9.6)	0.612 (0.551, 0.693)	<0.001
2019	2,466 (8.4)	1,300 (8.8)	1,166 (7.9)	0.633 (0.562, 0.713)	<0.001
2020	2,093 (7.1)	1,128 (7.6)	965 (6.5)	0.604 (0.533, 0.683)	<0.001
2021	1,777 (6.0)	1,041 (7.1)	736 (5.0)	0.499 (0.438, 0.568)	<0.001
2022	1,169 (4.0)	703 (4.8)	466 (3.2)	0.468 (0.404, 0.54)	<0.001
Hysterectomy for benign disease	9,360 (31.8)	4,998 (33.9)	4,362 (29.6)	0.819 (0.78, 0.860)	<0.001
Methods of surgery for endometriosis					
Ovarian cystectomy	18,393 (62.3)	9,041 (61.3)	9,352 (63.4)	1.094 (1.044,1.147)	<0.001
BSO or USO	3,660 (12.4)	1,790 (12.1)	1,870 (12.7)	1.051 (0.981,1.127)	0.158
Hysterectomy	9,308 (31.5)	4,932 (33.4)	4,376 (29.7)	0.84 (0.8,0.882)	<0.001
Fulguration	976 (3.3)	458 (3.1)	518 (3.5)	1.136 (1.000,1.291)	0.051
Number of surgery for endometriosis					
1	28,495 (96.5)	14,366 (97.3)	14,129 (95.7)	ref	
2	976 (3.3)	379 (2.6)	597 (4.0)	1.602 (1.405, 1.825)	<0.001
Over 3	47 (0.2)	14 (0.1)	33 (0.2)	2.397 (1.283, 4.478)	0.006
Time between endometriosis diagnosis and beginning of HRT					
Rate of HRT use before endometriosis diagnosis			3,862 (26.2)		
Time of HRT use before endometriosis diagnosis (years)			0.7 ± 1.7		
Time of HRT use after endometriosis diagnosis (years)			1.7 ±2.1		
Rate of HRT use after endometriosis diagnosis			10,862 (73.8)		
Time of HRT use after endometriosis diagnosis (years)			1.2 ±1.9		

CI, confidence interval; HIRA, Health Insurance Review & Assessment Service; HRT, hormone replacement therapy; OR, odds ratio.
All values are expressed as mean ± standard deviation or number (%).
[a] Univariate logistic regression

G. Dataset for liver cancer.

	Total n = 29,466 (100.0%)	HRT (-) n = 14,733 (50.0%)	HRT (+) n = 14,733 (50.0%)	OR (95% CI)	*P* value [a]
Age at last clinic visit (years)	55.5 ± 4.9	55.5 ±4.9	55.5 ±4.9	1.000 (0.995, 1.005)	1.000
SES at last clinic visit					
Mid- or high-SES	28,686 (97.4)	14,361 (97.5)	14,325 (97.2)	ref	
Low SES	780 (2.7)	372 (2.5)	408 (2.8)	1.1 (0.954, 1.268)	0.192
CCI at last clinic visit					
0	15,683 (53.2)	8,080 (54.8)	7,603 (51.6)	ref	
1	7,092 (24.1)	3,374 (22.9)	3,718 (25.2)	1.171 (1.107, 1.239)	<0.001
2	3,766 (12.8)	1,861 (12.6)	1,905 (12.9)	1.088 (1.013, 1.168)	0.020
3	1,552 (5.3)	733 (5.0)	819 (5.6)	1.187 (1.07, 1.318)	0.001
Over 4	1,373 (4.7)	685 (4.7)	688 (4.7)	1.067 (0.956, 1.192)	0.247
Age at endometriosis diagnosis (years)	48.4 ± 6.2	48.8 ± 6.1	48.0 ± 6.2	0.979 (0.976, 0.983)	<0.001
Year of endometriosis diagnosis					
2008	1,981 (6.7)	826 (5.6)	1,155 (7.8)	ref	
2009	1,886 (6.4)	879 (6.0)	1,007 (6.8)	0.819 (0.722, 0.930)	0.002
2010	1,928 (6.5)	829 (5.6)	1,099 (7.5)	0.948 (0.835, 1.076)	0.410
2011	1,697 (5.8)	793 (5.4)	904 (6.1)	0.815 (0.715, 0.929)	0.002
2012	1,512 (5.1)	700 (4.8)	812 (5.5)	0.83 (0.725, 0.949)	0.007
2013	1,824 (6.2)	879 (6.0)	945 (6.4)	0.769 (0.676, 0.874)	<0.001
2014	2,130 (7.2)	1,000 (6.8)	1,130 (7.7)	0.808 (0.714, 0.914)	0.001
2015	1,794 (6.1)	901 (6.1)	893 (6.1)	0.709 (0.623, 0.806)	<0.001
2016	1,701 (5.8)	880 (6.0)	821 (5.6)	0.667 (0.586, 0.760)	<0.001
2017	2,517 (8.5)	1,293 (8.8)	1,224 (8.3)	0.677 (0.601, 0.762)	<0.001
2018	3,050 (10.4)	1,629 (11.1)	1,421 (9.7)	0.624 (0.557, 0.699)	<0.001
2019	2,476 (8.4)	1,313 (8.9)	1,163 (7.9)	0.634 (0.562, 0.714)	<0.001
2020	2,074 (7.0)	1,114 (7.6)	960 (6.5)	0.616 (0.544, 0.698)	<0.001
2021	1,741 (5.9)	1,005 (6.8)	736 (5)	0.524 (0.46, 0.597)	<0.001
2022	1,155 (3.9)	692 (4.7)	463 (3.1)	0.479 (0.413, 0.555)	<0.001
Hysterectomy for benign disease	9,369 (31.8)	5,008 (34.0)	4,361 (29.6)	0.817 (0.777, 0.858)	<0.001
Methods of surgery for endometriosis					
Ovarian cystectomy	18,338 (62.2)	9,003 (61.1)	9,335 (63.4)	1.101 (1.050, 1.154)	<0.001
BSO or USO	3,629 (12.3)	1,752 (11.9)	1,877 (12.7)	1.082 (1.009, 1.16)	0.027
Hysterectomy	9,371 (31.8)	5,010 (34.0)	4,361 (29.6)	0.816 (0.777, 0.857)	<0.001
Fulguration	9,70 (3.3)	456 (3.1)	514 (3.5)	1.132 (0.996, 1.287)	0.058
Number of surgery for endometriosis					
1	28,459 (96.6)	14,355 (97.4)	14,104 (95.7)	ref	
2	966 (3.3)	368 (2.5)	598 (4.1)	1.654 (1.45, 1.887)	<0.001
Over 3	41 (0.1)	10 (0.1)	31 (0.2)	3.155 (1.546, 6.438)	0.002
Time between endometriosis diagnosis and beginning of HRT					
Rate of HRT use before endometriosis diagnosis			3,846 (26.1)		
Time of HRT use before endometriosis diagnosis (years)			0.7 ±1.7		
Time of HRT use after endometriosis diagnosis (years)			1.7 ± 2.1		
Rate of HRT use after endometriosis diagnosis			10,887 (73.9)		
Time of HRT use after endometriosis diagnosis (years)			1.3 ± 1.9		

CI, confidence interval; HIRA, Health Insurance Review & Assessment Service; HRT, hormone replacement therapy; OR, odds ratio.
All values are expressed as mean ± standard deviation or number (%).
[a] Univariate logistic regression

H. Dataset for lung cancer.

	Total n = 29,624 (100.0%)	HRT (-) n = 14,812 (50.0%)	HRT (+) n = 14,812 (50.0%)	OR (95% CI)	*P* value [a]
Age at last clinic visit (years)	55.5 ± 4.9	55.5 ± 4.9	55.5 ± 4.9	1.000 (0.995, 1.005)	1.000
SES at last clinic visit					
Mid- or high-SES	28,839 (97.4)	14,442 (97.5)	14,397 (97.2)	ref	
Low SES	78 5(2.7)	370 (2.5)	415 (2.8)	1.125 (0.976, 1.297)	0.104
CCI at last clinic visit					
0	15,705 (53.0)	8,072 (54.5)	7,633 (51.5)	ref	
1	7,141 (24.1)	3,402 (23.0)	3,739 (25.2)	1.162 (1.099, 1.229)	<0.001
2	3,780 (12.8)	1,863 (12.6)	1,917 (12.9)	1.088 (1.014, 1.168)	0.02
3	1,589 (5.4)	759 (5.1)	830 (5.6)	1.156 (1.043, 1.282)	0.006
Over 4	1,409 (4.8)	716 (4.8)	693 (4.7)	1.024 (0.918, 1.142)	0.676
Age at endometriosis diagnosis (years)	48.5 ± 6.2	48.9 ± 6.1	48.0 ± 6.2	0.979 (0.975, 0.982)	<0.001
Year of endometriosis diagnosis					
2008	1,995 (6.7)	837 (5.7)	1,158 (7.8)	ref	
2009	1,885 (6.4)	869 (5.9)	1,016 (6.9)	0.845 (0.744, 0.959)	0.009
2010	1,920 (6.5)	818 (5.5)	1,102 (7.4)	0.974 (0.858, 1.105)	0.681
2011	1,670 (5.6)	759 (5.1)	911 (6.2)	0.868 (0.761, 0.989)	0.034
2012	1,520 (5.1)	705 (4.8)	815 (5.5)	0.836 (0.731, 0.956)	0.009
2013	1,857 (6.3)	902 (6.1)	955 (6.5)	0.765 (0.674, 0.869)	<0.001
2014	2,157 (7.3)	1,023 (6.9)	1,134 (7.7)	0.801 (0.709, 0.906)	<0.001
2015	1,825 (6.2)	929 (6.3)	896 (6.1)	0.697 (0.614, 0.792)	<0.001
2016	1,701 (5.7)	875 (5.9)	826 (5.6)	0.682 (0.599, 0.777)	<0.001
2017	2,532 (8.6)	1,306 (8.8)	1,226 (8.3)	0.679 (0.603, 0.764)	<0.001
2018	3,011 (10.2)	1,577 (10.7)	1,434 (9.7)	0.657 (0.586, 0.737)	<0.001
2019	2,460 (8.3)	1,297 (8.8)	1,163 (7.9)	0.648 (0.575, 0.730)	<0.001
2020	2,090 (7.1)	1,121 (7.6)	969 (6.5)	0.625 (0.552, 0.707)	<0.001
2021	1,814 (6.1)	1,075 (7.3)	739 (5.0)	0.497 (0.437, 0.565)	<0.001
2022	1,187 (4.0)	719 (4.9)	468 (3.2)	0.471 (0.406, 0.545)	<0.001
Hysterectomy for benign disease	9,382 (31.7)	4,992 (33.7)	4,390 (29.6)	0.829 (0.789, 0.870)	<0.001
Methods of surgery for endometriosis					
Ovarian cystectomy	18,431 (62.2)	9,046 (61.1)	9,385 (63.4)	1.102 (1.052, 1.155)	<0.001
BSO or USO	3,671 (12.4)	1,788 (12.1)	1,883 (12.7)	1.061 (0.990, 1.137)	0.094
Hysterectomy	9,382 (31.7)	4992 (33.7)	4,390 (29.6)	0.829 (0.789, 0.870)	<0.001
Fulguration	978 (3.3)	461 (3.1)	517 (3.5)	1.126 (0.991, 1.279)	0.069
Number of surgery for endometriosis					
1	28,570 (96.4)	14,391 (97.2)	14,179 (95.7)	ref	
2	1006 (3.4)	406 (2.7)	600 (4.1)	1.5 (1.32, 1.705)	<0.001
Over 3	48 (0.2)	15 (0.1)	33 (0.2)	2.233 (1.213, 4.111)	0.01
Time between endometriosis diagnosis and beginning of HRT					
Rate of HRT use before endometriosis diagnosis			3,868 (26.1)		
Time of HRT use before endometriosis diagnosis (years)			0.7 ± 1.7		
Time of HRT use after endometriosis diagnosis (years)			1.7 ± 2.1		
Rate of HRT use after endometriosis diagnosis			10,944 (73.9)		
Time of HRT use after endometriosis diagnosis (years)			1.3 ± 1.9		

CI, confidence interval; HIRA, Health Insurance Review & Assessment Service; HRT, hormone replacement therapy; OR, odds ratio.
All values are expressed as mean ± standard deviation or number (%).
[a] Univariate logistic regression

I. Dataset for pancreatic cancer.

	Total<br>n = 29,448<br>(100.0%)	HRT (-)<br>n = 14,724<br>(50.0%)	HRT (+)<br>n = 14,724<br>(50.0%)	OR (95% CI)	*P* value [a]
Age at last clinic visit (years)	55.5 ± 4.9	55.5 ± 4.9	55.5 ± 4.9	1.000 (0.995, 1.005)	1.000
SES at last clinic visit					
Mid- or high-SES	28,675 (97.4)	14,362 (97.5)	14,313 (97.2)	ref	
Low SES	773 (2.6)	362 (2.5)	411 (2.8)	1.139 (0.987, 1.315)	0.074
CCI at last clinic visit					
0	15,631 (53.1)	8,032 (54.6)	7,599 (51.6)	ref	
1	7,075 (24.0)	3,362 (22.8)	3,713 (25.2)	1.167 (1.104, 1.235)	<0.001
2	3,777 (12.8)	1,878 (12.8)	1,899 (12.9)	1.069 (0.996, 1.148)	0.067
3	1,586 (5.4)	764 (5.2)	822 (5.6)	1.137 (1.026, 1.261)	0.015
Over 4	1,379 (4.7)	688 (4.7)	691 (4.7)	1.062 (0.951, 1.185)	0.287
Age at endometriosis diagnosis (years)	48.5 ± 6.2	48.9 ± 6.1	48.0 ± 6.2	0.978 (0.975, 0.982)	<0.001
Year of endometriosis diagnosis					
2008	1,966 (6.7)	814 (5.5)	1,152 (7.8)	ref	
2009	1,877 (6.4)	861 (5.9)	1,016 (6.9)	0.834 (0.734, 0.947 3)	0.005
2010	1,917 (6.5)	821 (5.6)	1,096 (7.4)	0.943 (0.830, 1.072)	0.369
2011	1,692 (5.8)	788 (5.4)	904 (6.1)	0.811 (0.711, 0.924)	0.002
2012	1,525 (5.2)	716 (4.9)	809 (5.5)	0.798 (0.698, 0.914)	0.001
2013	1,831 (6.2)	881 (6.0)	950 (6.5)	0.762 (0.670, 0.866)	<0.001
2014	2,.122 (7.2)	996 (6.8)	1,126 (7.7)	0.799 (0.706, 0.904)	<0.001
2015	1,806 (6.1)	912 (6.2)	894 (6.1)	0.693 (0.609, 0.788)	<0.001
2016	1,677 (5.7)	858 (5.8)	819 (5.6)	0.675 (0.592, 0.769)	<0.001
2017	2,516 (8.5)	1,294 (8.8)	1,222 (8.3)	0.667 (0.592, 0.752)	<0.001
2018	2,973 (10.1)	1,551 (10.5)	1,422 (9.7)	0.648 (0.577, 0.727)	<0.001
2019	2,498 (8.5)	1,345 (9.1)	1,153 (7.8)	0.606 (0.538, 0.683)	<0.001
2020	2,126 (7.2)	1,167 (7.9)	959 (6.5)	0.581 (0.513, 0.657)	<0.001
2021	1,744 (5.9)	1,009 (6.9)	735 (5.0)	0.515 (0.452, 0.587)	<0.001
2022	1,178 (4)	711 (4.8)	467 (3.2)	0.464 (0.401, 0.538)	<0.001
Hysterectomy for benign disease	9,353 (31.7)	4,998 (33.9)	4,355 (29.6)	0.817 (0.778, 0.859)	<0.001
Methods of surgery for endometriosis					
Ovarian cystectomy	18,299 (62.1)	8,964 (60.9)	9,335 (63.4)	1.113 (1.062, 1.167)	<0.001
BSO or USO	3,627 (12.3)	1,758 (11.9)	1,869 (12.7)	1.072 (1.000, 1.15)	0.049
Hysterectomy	9,354 (31.8)	4,999 (34.0)	4,355 (29.6)	0.817 (0.778, 0.858)	<0.001
Fulguration	978 (3.3)	461 (3.1)	517 (3.5)	1.126 (0.991, 1.279)	0.069
Number of surgery for endometriosis					
1	28,438 (96.6)	14,338 (97.4)	14,100 (95.8)	ref	
2	966 (3.3)	374 (2.5)	592 (4.0)	1.61 (1.411, 1.836)	<0.001
Over 3	44 (0.2)	12 (0.1)	32 (0.2)	2.712 (1.397, 5.264)	0.003
Time between endometriosis diagnosis and beginning of HRT					
Rate of HRT use before endometriosis diagnosis			3,848 (26.1)		
Time of HRT use before endometriosis diagnosis (years)			0.7 ± 1.7		
Time of HRT use after endometriosis diagnosis (years)			1.7 ± 2.1		
Rate of HRT use after endometriosis diagnosis			10,876 (73.9)		
Time of HRT use after endometriosis diagnosis (years)			1.3 ± 1.9		

CI, confidence interval; HIRA, Health Insurance Review & Assessment Service; HRT, hormone replacement therapy; OR, odds ratio.
All values are expressed as mean ± standard deviation or number (%).
[a] Univariate logistic regression

J. Dataset for thyroid cancer.

	Total n = 28,356 (100.0%)	HRT (-) n = 14,178 (50.0%)	HRT (+) n = 14,178 (50.0%)	OR (95% CI)	*P* value [a]
Age at last clinic visit (years)	55.5 ± 4.9	55.5 ± 4.9	55.5 ± 4.9	1.000 (0.995, 1.005)	1.000
SES at last clinic visit					
Mid- or high-SES	27,612 (97.4)	13,833 (97.6)	13,779 (97.2)	ref	
Low SES	744 (2.6)	345 (2.4)	399 (2.8)	1.161 (1.003, 1.344)	0.045
CCI at last clinic visit					
0	15,311 (54)	7,878 (55.6)	7,433 (52.4)	ref	
1	6,915 (24.4)	3,271 (23.1)	3,644 (25.7)	1.181 (1.116, 1.25)	<0.001
2	3,448 (12.2)	1,713 (12.1)	1,735 (12.2)	1.074 (0.997, 1.156)	0.060
3	1,425 (5.0)	677 (4.8)	748 (5.3)	1.171 (1.050, 1.306)	0.004
Over 4	1,257 (4.4)	639 (4.5)	618 (4.4)	1.025 (0.914, 1.150)	0.674
Age at endometriosis diagnosis (years)	48.4 ± 6.2	48.9 ± 6.2	48.0 ± 6.2	0.977 (0.974, 0.981)	<0.001
Year of endometriosis diagnosis					
2008	1,939 (6.8)	811 (5.7)	1,128 (8.0)	ref	
2009	1,812 (6.4)	821 (5.8)	991 (7.0)	0.868 (0.763, 0.988)	0.032
2010	1,868 (6.6)	808 (5.7)	1,060 (7.5)	0.943 (0.829, 1.073)	0.373
2011	1,646 (5.8)	761 (5.4)	885 (6.2)	0.836 (0.732, 0.955)	0.008
2012	1,439 (5.1)	659 (4.7)	780 (5.5)	0.851 (0.742, 0.976)	0.021
2013	1,743 (6.2)	833 (5.9)	910 (6.4)	0.785 (0.69, 0.895)	<0.001
2014	2,046 (7.2)	958 (6.8)	1,088 (7.7)	0.817 (0.720, 0.926)	0.002
2015	1,750 (6.2)	888 (6.3)	862 (6.1)	0.698 (0.613, 0.795)	<0.001
2016	1,639 (5.8)	856 (6.0)	783 (5.5)	0.658 (0.576, 0.751)	<0.001
2017	2,390 (8.4)	1,214 (8.6)	1,176 (8.3)	0.697 (0.617, 0.786)	<0.001
2018	2,858 (10.1)	1,483 (10.5)	1,375 (9.7)	0.667 (0.593, 0.749)	<0.001
2019	2,402 (8.5)	1,288 (9.1)	1,114 (7.9)	0.622 (0.551, 0.702)	<0.001
2020	2,037 (7.2)	1,137 (8.0)	900 (6.4)	0.569 (0.502, 0.645)	<0.001
2021	1,656 (5.8)	970 (6.8)	686 (4.8)	0.509 (0.445, 0.581)	<0.001
2022	1,131 (4.0)	691 (4.9)	440 (3.1)	0.458 (0.394, 0.532)	<0.001
Hysterectomy for benign disease	8,923 (31.5)	4,775 (33.7)	4,148 (29.3)	0.814 (0.775, 0.856)	<0.001
Methods of surgery for endometriosis					
Ovarian cystectomy	17,630 (62.2)	8,620 (60.8)	9010(63.6)	1.124 (1.071, 1.179)	<0.001
BSO or USO	3,568 (12.6)	1,751 (12.4)	1817(12.8)	1.043 (0.973, 1.119)	0.237
Hysterectomy	8,928 (31.5)	4,779 (33.7)	4149(29.3)	0.814 (0.774, 0.856)	<0.001
Fulguration	944 (3.3)	450 (3.2)	494(3.5)	1.101 (0.967, 1.254)	0.145
Number of surgery for endometriosis					
1	27,370 (96.5)	13,800 (97.3)	13,570 (95.7)	ref	
2	946 (3.3)	370 (2.6)	576 (4.1)	1.583 (1.386, 1.808)	<0.001
Over 3	40 (0.1)	8 (0.1)	32 (0.2)	4.068 (1.875, 8.827)	<0.001
Time between endometriosis diagnosis and beginning of HRT					
Rate of HRT use before endometriosis diagnosis			3,700 (26.1)		
Time of HRT use before endometriosis diagnosis (years)			0.7 ± 1.7		
Time of HRT use after endometriosis diagnosis (years)			1.7 ± 2.1		
Rate of HRT use after endometriosis diagnosis			10,478 (43.9)		
Time of HRT use after endometriosis diagnosis (years)			1.3 ± 1.9		

CI, confidence interval; HIRA, Health Insurance Review & Assessment Service; HRT, hormone replacement therapy; OR, odds ratio.
All values are expressed as mean ± standard deviation or number (%).
[a] Univariate logistic regression
