# Supplementary material for: Hormone Replacement Therapy and Risks of Various Cancers in Postmenopausal Women with De Novo or a History of Endometriosis
Source: Cancers (Basel). 2024 Feb 16;16(4):809. doi: 10.3390/cancers16040809 (PMC10886569; doi:10.3390/cancers16040809)
Supplement: Supplementary file 1 [file cancers-16-00809-s001.zip › Table S3.pdf]

Table S3. Associations between the risk factors and various cancer occurrences according to HRT in women with postmenopausal endometriosis (HIRA claims data 2008–2022).

	Total		HRT (-)		HRT (+)	
	HR (95% CI) [a]	*P* value [a]	HR (95% CI) [b]	*P* value [b]	HR (95% CI) [c]	*P* value [c]
Age at endometriosis diagnosis per 5 years (years)						
Cervical cancer	1.023 (0.989, 1.058)	0.186	1.048 (1.006, 1.093)	0.025	0.985 (0.929, 1.045)	0.621
Uterine cancer	1.023 (0.999, 1.047)	0.063	1.021 (0.988, 1.055)	0.218	1.028 (0.992, 1.065)	0.132
Ovarian cancer	1.034 (1.006, 1.063)	0.016	1.045 (1.008, 1.083)	0.016	1.013 (0.968, 1.060)	0.585
Breast cancer	0.974 (0.956, 0.993)	0.007	0.965 (0.937, 0.993)	0.013	0.983 (0.957, 1.009)	0.203
Colon cancer	1.040 (1.010, 1.071)	0.008	1.028 (0.983, 1.075)	0.225	1.047 (1.004, 1.092)	0.033
Gastric cancer	1.042 (1.003, 1.082)	0.033	1.024 (0.969, 1.083)	0.402	1.049 (0.993, 1.107)	0.086
Liver cancer	1.017 (0.99, 1.046)	0.223	1.031 (0.993, 1.070)	0.110	1.000 (0.958, 1.044)	0.993
Lung cancer	1.055 (1.022, 1.09)	0.001	1.0476 (1.002, 1.096)	0.043	1.065 (1.017, 1.115)	0.007
Pancreatic cancer	1.011 (0.981, 1.042)	0.479	1.017 (0.974, 1.063)	0.445	1.001 (0.958, 1.045)	0.974
Thyroid cancer	0.987 (0.968, 1.006)	0.168	0.982 (0.954, 1.011)	0.226	0.991 (0.966, 1.018)	0.521
Year of endometriosis diagnosis						
Cervical cancer	1.233 (1.147, 1.325)	<0.001	1.279 (1.147, 1.426)	<0.001	1.225 (1.098, 1.367)	<0.001
Uterine cancer	1.299 (1.237, 1.365)	<0.001	1.344 (1.249, 1.447)	<0.001	1.260 (1.176, 1.350)	<0.001
Ovarian cancer	1.208 (1.139, 1.281)	<0.001	1.148 (1.062, 1.241)	<0.001	1.298 (1.178, 1.413)	<0.001
Breast cancer	1.012 (0.977, 1.049)	0.506	0.984 (0.937, 1.034)	0.529	1.032 (0.979, 1.088)	0.236
Colon cancer	0.914 (0.854, 0.979)	0.01	0.869 (0.787, 0.96)	0.005	0.965 (0.877, 1.062)	0.466
Gastric cancer	0.892 (0.818, 0.973)	0.010	0.916 (0.818, 1.026)	0.128	0.868 (0.756, 0.998)	0.046
Liver cancer	0.926 (0.872, 0.985)	0.014	0.886 (0.818, 0.961)	0.003	0.976 (0.888, 1.072)	0.604
Lung cancer	0.895 (0.828, 0.967)	0.005	0.906 (0.818, 1.003)	0.056	0.883 (0.782, 0.997)	0.044
Pancreatic cancer	0.967 (0.907, 1.031)	0.299	0.929 (0.851, 1.014)	0.100	1.004 (0.914, 1.104)	0.927
Thyroid cancer	0.99 (0.954, 1.028)	0.597	0.971 (0.921, 1.024)	0.274	0.995 (0.944, 1.049)	0.852
Number of surgery for endometriosis						
Cervical cancer	2.3 (0.968, 5.462)	0.059	1.280 (0.181, 9.047)	0.805	2.578 (0.976, 6.812)	0.056
Uterine cancer	2.938 (1.751, 4.93)	<0.001	2.458 (0.930, 6.495)	0.07	3.345 (1.800, 6.215)	<0.001
Ovarian cancer	3.253 (1.755, 6.031)	<0.001	3.127 (1.166, 8.386)	0.024	3.272 (1.473, 7.266)	0.004
Breast cancer	1.305 (0.873, 1.949)	0.195	1.361 (0.745, 2.485)	0.316	1.289 (0.752, 2.21)	0.356
Colon cancer	1.542 (0.702, 3.386)	0.281	0.692 (0.100, 4.782)	0.709	2.089 (0.879, 4.963)	0.095
Gastric cancer [d]	0.441 (0.063, 3.093)	0.410	.	.	0.736 (0.106, 5.122)	0.757
Liver cancer	0.392 (0.098, 1.561)	0.184	0.493 (0.07, 3.47)	0.477	0.324 (0.046, 2.288)	0.258
Lung cancer	2.072 (0.943, 4.549)	0.07	2.473 (0.814, 7.512)	0.110	1.854 (0.602, 5.711)	0.282
Pancreatic cancer	0.906 (0.344, 2.389)	0.842	0.66 (0.095, 4.583)	0.674	1.061 (0.345, 3.261)	0.918
Thyroid cancer	1.707 (1.175, 2.481)	0.005	1.157 (0.518, 2.588)	0.722	1.987 (1.303, 3.03)	0.001
Hysterectomy for benign disease						
Cervical cancer	0.145 (0.075, 0.281)	<0.001	0.131 (0.056, 0.306)	<0.001	0.205 (0.071, 0.587)	0.003
Uterine cancer	0.03 (0.014, 0.065)	<0.001	0.007 (0.001, 0.054)	<0.001	0.06 (0.025, 0.143)	<0.001
Ovarian cancer	0.168 (0.1, 0.285)	<0.001	0.158 (0.076, 0.327)	<0.001	0.185 (0.086, 0.397)	<0.001
Breast cancer	0.793 (0.573, 1.096)	0.16	0.766 (0.489, 1.202)	0.246	0.879 (0.548, 1.411)	0.594
Colon cancer	0.758 (0.385, 1.492)	0.423	1.046 (0.402, 2.721)	0.926	0.546 (0.206, 1.448)	0.224
Gastric cancer	0.739 (0.303, 1.802)	0.506	0.413 (0.117, 1.454)	0.169	1.429 (0.374, 5.452)	0.602
Liver cancer	0.849 (0.471, 1.528)	0.584	0.837 (0.382, 1.834)	0.657	0.924 (0.375, 2.273)	0.862
Lung cancer	0.718 (0.325, 1.584)	0.411	0.742 (0.280, 1.966)	0.549	0.665 (0.168, 2.625)	0.560
Pancreatic cancer	0.77 (0.425, 1.396)	0.389	0.659 (0.284, 1.53)	0.331	0.952 (0.405, 2.237)	0.911
Thyroid cancer	0.788 (0.557, 1.115)	0.178	0.774 (0.477, 1.257)	0.301	0.842 (0.511, 1.389)	0.501

CI, confidence interval; HIRA, health insurance review & assessment service; HR, hazard ratio; HRT, hormone replacement therapy.
[a] The data were adjusted for age at endometriosis diagnosis, year of endometriosis diagnosis, number of surgery for endometriosis, hysterectomy for benign disease, and HRT.
[b] The data were adjusted for age at endometriosis diagnosis, year of endometriosis diagnosis, number of surgery for endometriosis, and hysterectomy for benign disease.
[c] The data were adjusted for age at endometriosis diagnosis, year of endometriosis diagnosis, number of surgery for endometriosis, hysterectomy for benign disease, duration of HRT and types of HRT medication.
[d] With relation to number of surgery for endometriosis, there were no women with gastric cancer in control group.
