# Supplementary material for: Hormone Replacement Therapy and Risks of Various Cancers in Postmenopausal Women with De Novo or a History of Endometriosis
Source: Cancers (Basel). 2024 Feb 16;16(4):809. doi: 10.3390/cancers16040809 (PMC10886569; doi:10.3390/cancers16040809)
Supplement: Supplementary file 1 [file cancers-16-00809-s001.zip › Table S4.pdf]

Table S4. Time between an endometriosis diagnosis and various cancer diagnoses (years) (HIRA claims data 2008–2022).

	Total (100.0%)	HRT (-) (50.0%)	HRT (+) (50.0%)	*P* value
Cervical cancer	6.7 ± 4.3	8.1 ± 5.5	5.5 ± 3.8	0.007
Uterine cancer	6.6 ± 3.8	6.3 ± 3.5	6.8 ± 4.0	0.373
Ovarian cancer	6.7 ± 3.8	6.7 ± 3.9	6.7 ± 3.8	0.969
Breast cancer	5.6 ± 3.4	5.8 ± 3.6	5.4 ± 3.3	0.285
Colon cancer	6.9 ± 4.1	7.2 ± 4.4	6.7 ± 3.8	0.535
Gastric cancer	6.3 ± 3.9	7.0 ± 3.9	5.7 ± 3.8	0.181
Liver cancer	6.8 ± 3.8	6.9 ± 3.8	6.6 ± 3.8	0.633
Lung cancer	7.1 ± 3.7	7.2 ± 3.8	6.9 ± 3.7	0.750
Pancreatic cancer	6.5 ± 3.8	6.6 ± 3.8	6.3 ± 3.8	0.695
Thyroid cancer	5.5 ± 3.5	6.0 ± 3.4	5.1 ± 3.4	0.014

CI, confidence interval; HIRA, health insurance review & assessment service; HR, hazard ratio; HRT, hormone replacement therapy.
Values are expressed as mean ± standard deviation.
