# Supplementary material for: Hormone Replacement Therapy and Risks of Various Cancers in Postmenopausal Women with De Novo or a History of Endometriosis
Source: Cancers (Basel). 2024 Feb 16;16(4):809. doi: 10.3390/cancers16040809 (PMC10886569; doi:10.3390/cancers16040809)
Supplement: Supplementary file 1 [file cancers-16-00809-s001.zip › Table S5.pdf]

Table S5. Time between the beginning of HRT and various cancer diagnoses (years) (HIRA claims data 2008–2022).

	HRT (+)
Cervical cancer	5.5 ± 3.8
Uterine cancer	6.8 ± 4.0
Ovarian cancer	6.7 ± 3.8
Breast cancer	5.4 ± 3.3
Colon cancer	6.7 ± 3.8
Gastric cancer	5.7 ± 3.8
Liver cancer	6.6 ± 3.8
Lung cancer	6.9 ± 3.7
Pancreatic cancer	6.3 ± 3.8
Thyroid cancer	5.1 ± 3.4

HIRA, Health Insurance Review & Assessment Service; HRT, hormone replacement therapy
Values are expressed as mean ± standard deviation.
