# Supplementary material for: Hormone Replacement Therapy and Risks of Various Cancers in Postmenopausal Women with De Novo or a History of Endometriosis
Source: Cancers (Basel). 2024 Feb 16;16(4):809. doi: 10.3390/cancers16040809 (PMC10886569; doi:10.3390/cancers16040809)
Supplement: Supplementary file 1 [file cancers-16-00809-s001.zip › Table S6.pdf]

**Table S6. Duration of HRT use in women with various cancers (years) (HIRA claims data 2008–2022).**

| | HRT (+) |
|---|---|
| Cervical cancer | 0.9 ± 1.5 |
| Uterine cancer | 1.3 ± 2.3 |
| Ovarian cancer | 1.7 ± 2.4 |
| Breast cancer | 1.3 ± 1.9 |
| Colon cancer | 2.0 ± 2.4 |
| Gastric cancer | 2.7 ± 3.0 |
| Liver cancer | 1.7 ± 2.4 |
| Lung cancer | 1.7 ± 2.4 |
| Pancreatic cancer | 1.7 ± 2.7 |
| Thyroid cancer | 1.4 ± 2.1 |

HIRA, Health Insurance Review & Assessment Service; HRT, hormone replacement therapy
Values are expressed as mean ± standard deviation.
