# Supplementary material for: Hormone Replacement Therapy and Risks of Various Cancers in Postmenopausal Women with De Novo or a History of Endometriosis
Source: Cancers (Basel). 2024 Feb 16;16(4):809. doi: 10.3390/cancers16040809 (PMC10886569; doi:10.3390/cancers16040809)
Supplement: Supplementary file 1 [file cancers-16-00809-s001.zip › Table S7.pdf]

Table S7. Ages at various cancer diagnoses (years) (HIRA claims data 2008–2022).

	Total (100.0%)	HRT (-) (50.0%)	HRT (+) (50.0%)	OR (95% CI)	*P* value
Cervical cancer	50.7 ± 8.6	54.7 ± 8.2	47.0 ± 7.3	0.979 (0.976, 0.983)	<0.001
Uterine cancer	50.4 ± 8.2	51.4 ± 6.8	49.5 ± 9.3	0.979 (0.975, 0.982)	<0.001
Ovarian cancer	50.6 ± 8.0	51.2 ± 7.8	50.0 ± 8.2	0.979 (0.976, 0.983)	<0.001
Breast cancer	45.6 ± 5.7	46.0 ± 5.4	45.3 ± 6.0	0.979 (0.975, 0.983)	<0.001
Colon cancer	47.7 ± 7.4	48.1 ±7.9	47.3 ± 6.9	0.98 (0.976, 0.983)	<0.001
Gastric cancer	47.6 ± 7.3	47.8 ± 6.5	47.4 ± 8.0	0.979 (0.975, 0.982)	<0.001
Liver cancer	46.8 ± 5.7	48.0 ± 6.2	45.6 ± 5.0	0.98 (0.976, 0.983)	<0.001
Lung cancer	48.1 ± 9.2	49.1 ± 9.4	47.0 ± 9.1	0.979 (0.976, 0.983)	<0.001
Pancreatic cancer	46.9 ± 7.1	47.7 ± 6.2	46.3 ± 7.8	0.979 (0.975, 0.982)	<0.001
Thyroid cancer	45.7 ± 6.3	46.7 ± 5.8	45.0 ± 6.6	0.978 (0.975, 0.982)	<0.001

CI, confidence interval; HIRA, Health Insurance Review & Assessment Service; HRT, hormone replacement therapy; OR, odds ratio.
Values are expressed as mean ± standard deviation.
